# Wang-Bi Tablet Ameliorates DMM-Induced Knee Osteoarthritis through Suppressing the Activation of p38-MAPK and NF-*κ*B Signaling Pathways in Mice

**DOI:** 10.1155/2021/3930826

**Published:** 2021-08-13

**Authors:** Hui Li, Yan You, Bing Jiang, Haidong Li, Xiang Li, Wei Wu, Hong Cao, Xiaoyan Shen, Jun Zou

**Affiliations:** ^1^School of Kinesiology, Shanghai University of Sport, No. 399, Changhai Road, Yangpu, Shanghai 200438, China; ^2^Department of Pharmacology, School of Pharmaceutical Sciences, Fudan University, No. 826, Zhangheng Road, Pudong, Shanghai 201203, China

## Abstract

**Background:**

Traditional Chinese medicine (TCM) exhibits outstanding therapeutic effects on the treatment of osteoarthritis (OA). Wang-Bi tablets (WBTs) have been used in clinics to treat knee osteoarthritis (KOA) by alleviating joint swelling and paining, and thus, the quality of life in patients with KOA was improved. However, its underlying molecular mechanism of anti-inflammatory response remains unclear. Therefore, further investigation is required.

**Purpose:**

This study aimed to explore the function of WBT in KOA mice and uncover the possible molecular mechanisms. *Study Design*. A KOA model was constructed by destabilizing the medial meniscus (DMM). IL-1*β*-treated chondrocytes were used to investigate the precise mechanism in vitro.

**Methods:**

(1) C57BL/6 male mice (8-week-old) were divided into Model, Sham, WBT-L, WBT-M, and WBT-H groups. After intragastric administration of 0.5% CMC-Na or WBT for 4 weeks, inflammation and pathological change were analyzed by ELISA, RT-qPCR, hematoxylin and eosin (H & E) and safranine O staining. (2) Isolated chondrocytes were stimulated with IL-1*β* followed by WBT-containing serum treatment, and then, the expression of inflammatory cytokines was analyzed by ELISA and RT-qPCR. (3) The effects of WBT on inflammatory signaling cascades in mice knee joint and chondrocytes were detected by WB.

**Results:**

The results indicated that WBT could alleviate inflammation and prevent cartilage injury in KOA mice. Compared with 0.5% CMC-Na-treated mice, the serum glycosaminoglycans (GAG) level in WBT-treated mice was notably increased, while the proinflammatory cytokine interleukin- (IL-) 6 level was decreased. In addition, WBT treatment suppressed the activation of NF-*κ*B and p38 signaling pathways both in vivo and in vitro.

**Conclusion:**

WBT can effectively inhibit articular cartilage injury and inflammatory response in KOA mice. The protective role of WBT in mice KOA was a result of the downregulation of NF-*κ*B and p38-MAPK signal pathways.

## 1. Introduction

Osteoarthritis (OA) is a common health problem that is characterized by articular cartilage loss and joint tissue remodeling [1]. It is estimated that more than 350 million people are currently suffering from OA in the world according to the Global Burden of Disease Study 2019 [2]. Knee osteoarthritis (KOA) is the most common type of OA. As reported, the high incidence of KOA among the elderly contributes to the poor quality of life by letting them bear the pain of physical disability [3, 4]. Although the pathogenesis of OA is not yet fully distinct and has much controversies, lots of studies have confirmed that the essential inflammatory factors including TNF-*α*, IL-1*β*, and IL-6 are upregulated in the early stage of OA [5].

p38 and NF-*κ*B pathways are essential in the pathogenesis and progression of considerable human diseases, especially in OA [6, 7]. As reported, the blockage of p38-MAPK with p38 inhibitor could alleviate the degradation of bone and cartilage by suppressing chondrocyte apoptosis, downstream inflammatory cytokine secretion, and inflammatory cell recruitment [8]. Similarly, nuclear factor-*κ*B (NF-*κ*B) is an essential regulator for inflammatory factors such as IL-6, which could increase the secretion of cartilage catabolic enzymes including matrix metalloproteinases (MMPs), thus causing extracellular matrix (ECM) degradation [9, 10]. Therefore, it is meaningful to explore appropriate drugs to alleviate the inflammatory injury of chondrocytes when treating OA clinically.

Wang-Bi tablet (WBT), a Chinese herbal formula, is used to treat rheumatoid arthritis, joint swelling, and stiffness in clinics for several years, which contains 16 herbal medicines, including Radix Rehmanniae, Radix Rehmanniae Preparata, Radix aconiti lateralis preparata, Rhizoma Drynariae, Cassia Twig, Radix Dipsaci, Epimedium brevicornu Maxim, Rhizoma Cibotii Preparata, Carthami Flos, Radix Clematidis, Spina Gleditsiae, Radix Angelicae Pubescentis, Radix Saposhnikoviae, Radix Paeoniae Alba, Rhizoma Anemarrhenae, and goat bone [11]. Due to its prominent therapeutic effect with few side effects, WBT has become more and more popular in clinical practice. However, few studies have been carried out on its mechanisms [12, 13]. As a result, the pharmacological mechanism of WBT in KOA is largely unclear. Our research aimed to explore the anti-inflammatory influence and underlying mechanism of WBT in mice KOA.

## 2. Materials and Methods

### 2.1. Reagents

WBTs (SFDA approval number Z20044066) were provided by Shanghai Pharmaceuticals Holding Co., Ltd. To prepare oral suspension, WBTs were dissolved in sterilized 0.5% CMC-Na. Mouse IL-6 and GAG ELISA kits were obtained from BioLegend. Antibodies against p65, p-p65, p-IkB kinase (IkK) *α*/*β*, IkB*α*, p38, and p-p38 were purchased from Epitomics. Anti-*β*-actin antibody was obtained from Sigma-Aldrich.

### 2.2. Preparation and Quantity Control of WBT

WBT containing sixteen kinds of herbal medicines was prepared as described in our previous works. WBT used for the present experiments was from the same batch as in the previous report, and the quality of WBT was also determined by high-performance liquid chromatography fingerprinting analysis [14].

### 2.3. Animal Experiments

All mice were fed in a 25 ± 1°C temperature and 50 ± 5% humidity SPF environment with a 12 h light/dark cyclic schedule and free access to standard diet. All animal procedures were authorized by the ethics committee of Experimental Research, Fudan University.

The KOA model was constructed by DMM. In short, the medial collateral ligament and the medial meniscus of knee joints were cut off. Then, the patella was reduced and the wound was cleaned with normal saline. The capsule of knees was opened as a sham operation (Sham group, *n* = 8) and lavaged with 0.5% CMC-Na solvent. After DMM surgery, mice were administered intragastrically with 0.5% CMC-Na solvent (Mod, *n* = 8), low dose of WBT (0.20 g/kg/d, WBT-L, *n* = 8), medium dose of WBT (0.40 g/kg/d, WBT-M, *n* = 8), and high dose of WBT (0.60 g/kg/d, WBT-H, *n* = 8) for 4 weeks, respectively.

### 2.4. Preparation of Medicated Sera

Rats were administered with WBT solvent at a dose of 2.8 g/kg/day or 0.5% CMC-Na solution for 7 days. On day 8, the serum from overnight fasting mice was isolated. WBT-containing serum was mixed with normal rat serum at the ratios of 1 : 4, 4 : 6, and 6 : 4 and then added to a basic medium to prepare 10% serum-containing culture medium.

### 2.5. Primary Mice Chondrocyte Isolation and Treatment

The knee cartilages were isolated from new-born mice and digested with 0.1% collagenase II for 2 h, followed by 0.05% collagenase II for another 10 h. Chondrocytes then were collected and cultured in 10% FBS-containing medium. When 80% to 90% confluency was reached, chondrocytes were harvested and passaged. The chondrocytes used in the current study were the second passage. For IL-1*β*-treated groups, chondrocytes were treated with 10 ng/ml IL-1*β* for 6 h and further cultured in a medium containing 10% normal serum or medicated sera for 48 h [14].

### 2.6. Histological Analysis

The knee joint of mice was isolated and embedded in paraffin. After slicing, these paraffin sections were submitted to H&E staining and safranin O staining [15, 16]. The quantification of cartilage damages was then evaluated according to the Osteoarthritis Research Society International (OARSI) cartilage OA histology grading system [16, 17].

### 2.7. ELISA

ELISA was applied to analyze the levels of IL-6 and GAG in the serum. All procedures were performed as per our previously published research [15].

### 2.8. RT-qPCR

Total RNA was extracted from the knee cartilages and managed according to our previous research. The cDNA was prepared by reverse transcription and submitted to quantitative PCR (Q-PCR) analysis as per previous research [18]. Primers used in this study were synthesized by Huagene, and primer sequences are listed in Table S1.

### 2.9. Western Blot

Total proteins from the knee cartilages were prepared according to our previous study. After quantification, the samples were submitted to western blotting analysis referring to the standard protocol [15].

### 2.10. Statistics

All the numerical data were expressed as mean ± standard error of mean (SEM), and the experiment was repeated for no less than 3 times. The independent-samples *t* test and one-way ANOVA were performed by Statistical Program for Social Sciences 13 (SPSS 13). In detail, the independent-samples *t* test was used for observing the difference between two groups, while one-way ANOVA was applied for multiple comparisons. *P* < 0.05 was considered as the significance level.

## 3. Results

### 3.1. WBT Alleviated the Inflammation and Cartilage Injury in KOA Mice

**Figure 1:**
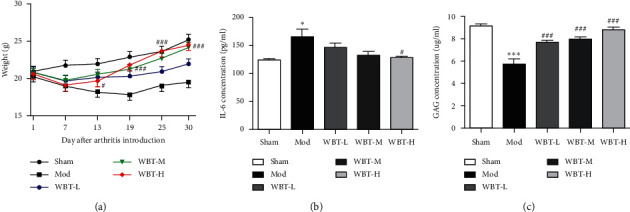
Therapeutic effect of WBT on KOA mice. Sham and Mod groups' mice administered by gavage with 0.5% CMC-Na solvent (Sham, Mod, *n* = 8), low dose of WBT (0.20 g/kg/d, WBT-L, *n* = 8), medium dose of WBT (0.40 g/kg/d, WBT-L, *n* = 8), and high dose of WBT (0.60 g/kg/d, WBT-H, *n* = 8) for 4 weeks, respectively. The body weight of each group was recorded. Effects of WBT on the IL-6 and GAG in KOA mice are given. The concentration of proinflammatory cytokine IL-6 (a) and GAG (b) in the serum was measured by ELISA. ^#^*P* < 0.05, ^##^*P* < 0.01, and ^###^*P* < 0.001 vs Mod group. ^*∗*^*P* < 0.05, ^*∗∗*^*P* < 0.01, and ^*∗∗∗*^*P* < 0.001 vs Sham group.

To evaluate the influence of WBT in osteoarthritis, we constructed a DMM-induced KOA model in mice and analyzed the degree of injury. As shown in Figure 1(a), WBT treatment significantly reversed the reduction of body weight compared with the Mod group. Inflammatory factors are important evaluation indexes for KOA, so the levels of various inflammatory factors in the serum were analyzed (Figure 1(b)). The level of proinflammatory cytokine IL-6 was significantly increased after DMM. However, high-dose WBT treatment notably reduced the production of IL-6 (Figure 1(b)). GAG, another key evaluation index for KOA, can stimulate the formation, repairment, and enhancement of the cartilage and promote the secretion of proteoglycan to lubricate the joint, thus improving joint pain and stiffness. As shown in Figure 1(c), the content of GAG in the Mod group was notably lower than that in the Sham group. Compared with the Mod group, GAG levels in WBT groups were notably increased in a dose-dependent manner. Together, these results suggested that WBT poses a repressive effect on the articular cartilage injury and inflammatory response in KOA mice.

### 3.2. WBT Improved the Injury of Joint Cartilage in KOA Mice

**Figure 2:**
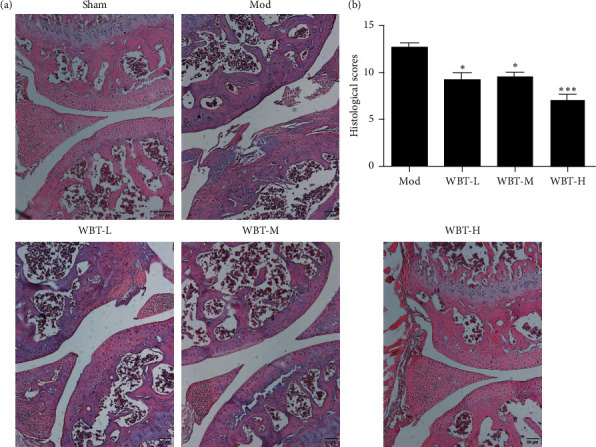
Effects of WBT on pathological changes of KOA mice. (a) The representative images of HE staining of the knee joint sections in each group. (b) The histological changes were scored and analyzed. ^*∗*^*P* < 0.05, ^*∗∗*^*P* < 0.01, and ^*∗∗∗*^*P* < 0.001 vs Mod group.

To further explore the possible role of WBT in the joint cartilage damages of KOA mice, the histological changes and inflammatory cytokine expression in the knee cartilages were measured. DMM could induce inflammatory cell infiltration and synoviocyte proliferation, which together contributed to the extensive pannus formation and serious cartilage destruction. However, after WBT administration, these phenomena were notably alleviated. The WBT-M and WBT-H groups only showed mild hyperplasia and few inflammatory cell infiltration (Figures 2(a) and 2(b)). In addition, the articular cartilage surface was smooth and the joint morphology was intact in these two groups.

**Figure 3:**
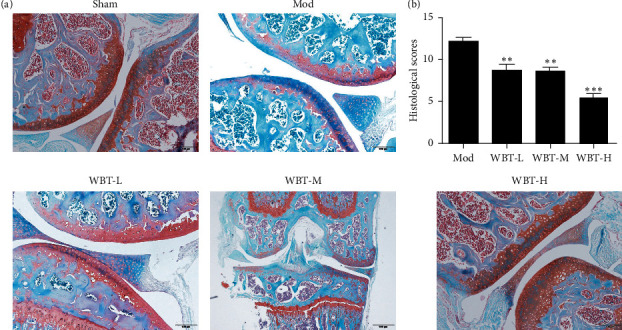
Effects of WBT on cartilage injury of KOA mice. (a) The representative images of safranin O and fast green staining of the knee joint section in each group. (b) The cartilage calcification changes were scored and analyzed. ^*∗*^*P* < 0.05, ^*∗∗*^*P* < 0.01, and ^*∗∗∗*^*P* < 0.001 vs Mod group.

Furthermore, safranin O and fast green examinations were performed by using mice knee joint sections. The knee joints of the Mod group exhibited tidal line damage and cartilage calcification, which was more serious than those in Sham group (Figures 3(a) and 3(b)). However, the cartilage injury and calcification were alleviated in all WBT groups. These results suggest that WBT can improve the cartilage injury KOA mice.

**Figure 4:**
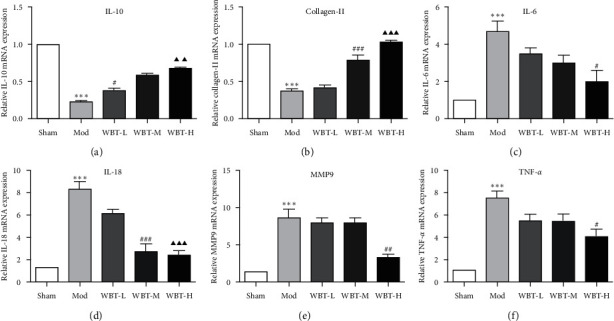
Effects of WBT on the expression of cytokine in the joints of KOA mice. (a–f) The mRNA levels of TNF-*α*, MMP9, IL-6, IL-18, IL-10, and collagen-II in the knee joints were analyzed by RT-qPCR. ^*∗∗∗*^*P* < 0.001 vs Sham group; ^#^*P* < 0.05, ^##^*P* < 0.01, and ^###^*P* < 0.001 vs Mod group; ^▲▲^*P* < 0.01 and ^▲▲▲^*P* < 0.001 vs WBT-L.

The relative cytokine mRNA levels from the knee cartilages were consistent with the histology. As seen, the anti-inflammatory cytokine IL-10 and collagen-II were notably increased in both WBT-M and WBT-H groups (Figures 4(a) and 4(b)). In contrast, IL-6, IL-18, MMP9, and TNF-*α* mRNA levels were obviously upregulated in the model group when compared with the Sham group. WBT treatment, to some extent, abolished DMM-induced proinflammatory cytokine expression (Figures 4(c)–4(f)). In summary, these findings indicate that WBT inhibited joint inflammation and cartilage injury in KOA mice.

### 3.3. WBT Attenuated the Phosphorylation of NF-*κ*B p65 and p38

**Figure 5:**
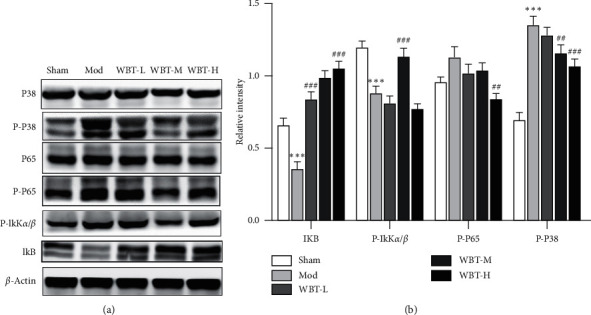
Effect of WBT on the phosphorylation of NF-*κ*B p65 and P38. In drug treatment groups, KOA mice were treated daily by gavage with WBT from the first day of arthritis onset for 4 weeks. Mice in Sham and Mod groups were given 0.5% CMC-Na solvent during the same period. (a) Total proteins from the knee joints of mice were isolated and subjected to immunoblotting with indicated antibodies. (b) Densitometry quantification in (a) was analyzed by ImageJ software. Data are expressed as means ± SEM. All experiments are repeated for three times at least. ^*∗*^*P* < 0.05, ^*∗∗*^*P* < 0.01, and ^*∗∗∗*^*P* < 0.001 vs Sham group; ^#^*P* < 0.05, ^##^*P* < 0.01, and ^###^*P* < 0.001 vs Mod group.

As known, the production of proinflammatory cytokines is associated with KOA progression. NF-*κ*B and p38 pathways are considered to induce excessive expression of proinflammatory cytokines. Therefore, we detected the activation of p38 and NF-*κ*B pathways in KOA mice (Figures 5(a) and 5(b)). As seen, WBT treatment reduced the phosphorylation of p65 and p38 in DMM-induced arthritis. These results suggested that WBT might suppress the inflammatory response in KOA via regulating the activation of these two signaling pathways mentioned above.

### 3.4. WBT Inhibits NF-*κ*B and p38 Signaling Pathways and Inflammation in IL-1*β*-Stimulated Chondrocytes

**Figure 6:**
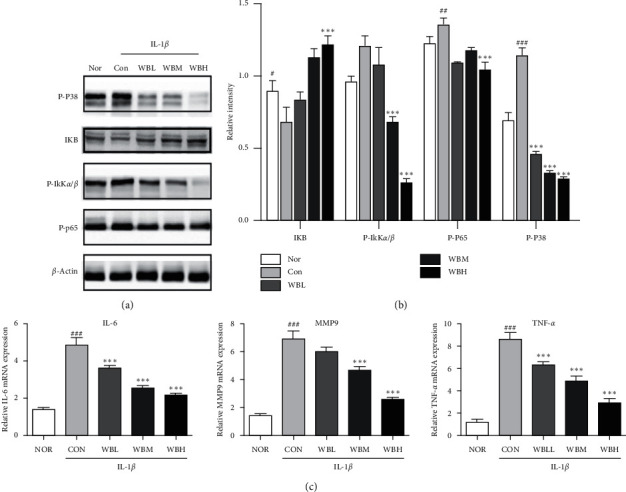
Effect of WBT on the phosphorylation of NF-*κ*B p65, p38 signaling pathways, and inflammation. Chondrocytes were treated with normal serum medium, WBT serum medium, WBL (low dose of WBT) serum medium, WBM (medium dose of WBT) serum medium, and WBH (high dose of WBT), followed by IL-1*β* stimulation. (a) Equal amount of proteins from the chondrocytes in each group were merged, and samples (30 *μ*g) were subjected to immunoblotting with indicated antibodies. (b) Densitometry quantification in (a) was analyzed by ImageJ software. Data are means ± SEM. All experiments are repeated for three times at least. (c) The mRNA levels of TNF-*α*, MMP9, and IL-6 were analyzed by RT-qPCR (a, b, c). ^#^*P* < 0.05, ^##^*P* < 0.01, and ^###^*P* < 0.001 vs Nor group; ^*∗*^*P* < 0.05, ^*∗∗*^*P* < 0.01, and ^*∗∗∗*^*P* < 0.001 vs Con group.

To further explore the role of WBT in NF-*κ*B and p38 signaling pathways in vitro, proteins from mice chondrocytes were isolated and the levels of I*κ*B, p-I*κ*K*α*/*β*, and p-p38 were detected. Figures 6(a) and 6(b) indicate that WBT-containing serum treatment could restrain the degradation of I*κ*B protein and reduce the level of p-I*κ*K*α*/*β* and p-p38. In addition, the increase of IL-6, MMP9, and TNF-*α* mRNA levels induced by IL-1*β* were notably suppressed by WBT-containing serum treatment in a dose-dependent manner (Figure 6(c)). All the above findings made it clear that the antiarthritic and anti-inflammatory effects of WBT are involved in the regulation of NF-*κ*B and p38-MAPK signaling pathways.

## 4. Discussion

Osteoarthritis (OA) is characterized by structural alterations in the whole joint including hyaline articular cartilage, subchondral bone, ligaments, capsule, synovium, and periarticular muscles [19, 20]. The high incidence of OA has become a major public health problem and attracted increasing attention. The pathogenesis of OA is unclear according to the present research. Moreover, the majority of patients with OA do not receive appropriate therapy [21]. As reported, education, self-management, and exercise are widely considered as first-line treatment. The most recommended pharmacological treatments are paracetamol and NSAID [22]. Unfortunately, these pharmacological treatments neither slow nor prevent OA progression. Therefore, it is of great interest to explore new effective therapeutic candidates that can strongly prevent cartilage injury along with less adverse effects. WBT is a traditional Chinese medicine that is widely used in herbal remedies in Asian countries [11, 23]. However, WBT's protective role in articular cartilage and related mechanisms in KOA chondrocytes have not been reported yet. In this study, we found that WBT could suppress KOA progression in the mice DMM model through inhibiting the p38 and NF-*κ*B pathways. Consistently, WBT attenuated the inflammatory response in chondrocytes via the same pathways.

Increasing expression of proteolytic enzymes such as MMPs is one of the important factors for OA development, which is crucial for the degradation of various ECM components of articular cartilage [24]. As reported, GAG can facilitate proteoglycan synthesis, alleviate the loss of cartilage matrix, and inhibit MMP9 expression, thus contributing to the repairment of the cartilage [25]. Due to its essential role in maintaining ECM homeostasis, GAG has been considered as an important indicator of cartilage injury in OA [26]. Here, we also found that WBT can increase the serum level of GAG in the mice KOA model, indicating that WBT probably alleviates articular cartilage injury by inhibiting the loss of GAG.

Previous studies indicated that NF-*κ*B signal pathway plays significant influence in OA pathogenesis and changing process [27]. The development of OA is accompanied by the excessive secretion of proinflammatory factors and the loss of collagen-II. Proinflammatory cytokines and other factors could activate NF-*κ*B pathway to attenuate the expression of large amount of cytokines and chemokines, such as IL-6 and TNF-*α* [28]. NF-*κ*B blockage can suppress IL-1*β*-induced inflammatory response, apoptosis, and collagen-II degradation in chondrocytes [29, 30]. WBT treatment significantly decreases the activity of NF-kB signaling pathway finally. Consistently, the levels of TNF-*α* mRNA and IL-6 mRNA in WBT-treated KOA mice were notably lower when compared to the model group, suggesting that WBT might inhibit inflammatory response and OA cartilage injury in the DMM mice model.

Mitogen-activated protein kinase (MAPK) signaling pathway-p38/JNK/ERK involves in the progression of OA, and p38-MAPK signaling pathway inhibition could alleviate the inflammation in KOA [8]. As shown, WBT treatment inhibited the phosphorylation of p38 and reduced the occurrence and development of inflammation. The inhibition of p38 and NF-kB pathways induced by WBT treatment might synergistically downregulate the proinflammatory factor expression and promote cartilage repair and regeneration, thereby achieving protective effect in KOA.

## 5. Conclusion

In this study, we proved that oral medication of WBT possesses antiarthritic effect on the KOA mice model. WBT treatment not only ameliorated cartilage injury and calcification but also inhibited inflammation response to improve poor functional outcome. Mechanically, WBT inhibits p38 and NF-*κ*B signaling pathways, thus suppressing the expression of proinflammatory cytokines. Altogether, the data acquired in the present study extend our understanding that WBT is a promising candidate for treating KOA.

## Data Availability

The data used to support the findings of this study are included within the article.

## Abbreviations

KOA: Knee osteoarthritis
OA: Osteoarthritis
WBT: Wang-Bi tablet
DMM: Destabilization of the medial meniscus
GAG: Glycosaminoglycans.

## Additional Points

*Highlights*. (i) Degenerative arthritis has a higher incidence than rheumatoid arthritis and significantly decreases patients' quality of life. (ii) Wang-Bi tablets possess prominent therapeutic effects on degenerative knee osteoarthritis in a dose-dependent manner. (iii) The outbreak of inflammation in degenerative knee osteoarthritis involves the activation of p38-MAPK and NF-*κ*B pathways, which can be suppressed by Wang-Bi tablet treatment.

## Supplementary Materials

Supplementary Materials Table S1: the primer sequences for RT-qPCR.
