# Supplementary material for: Wang-Bi Tablet Ameliorates DMM-Induced Knee Osteoarthritis through Suppressing the Activation of p38-MAPK and NF-κB Signaling Pathways in Mice
Source: Evid Based Complement Alternat Med. 2021 Aug 13;2021:3930826. doi: 10.1155/2021/3930826 (PMC8380173; doi:10.1155/2021/3930826)
Supplement: Supplementary Materials — Table S1: the primer sequences for RT-qPCR. [file 3930826.f1.docx]

**Table**

Table S1 The primer sequences for RT- qPCR

| **Gene** | **Primer sequence (5’-3’)** |
| --- | --- |
| **mβ-Actin** | Forward: 5’-CTGTCCCTGTATGCCTCTG-3’  Reverse: 5’-ATGTCACGCACGATTTCC-3’ |
| **mTNF-α** | Forward: 5’-AGGCTGCCCCGACTACGT-3’ |
|  | Reverse: 5’-GACTTTCTCCTGGTATGAGATAGCAAA-3’ |
| **mIL-18** | Forward: 5’-GGCCGACTTCACTGTACAACCG-3’ |
|  | Reverse: 5’-GGTCACAGCCAGTCCTCTTACTTC-3’ |
| **mIL-6** | Forward: 5’-ACTTCCATCCAGTTGCCTTCTTGG-3’ |
|  | Reverse: 5’-TTAAGCCTCCGACTTGTGAAGTGG-3’ |
| **mCollagenⅡ** | Forward: 5’-GTGAGCATGCAGCTTTTGCC-3’ |
|  | Reverse: 5’-GGGCCACGAGGTCAATGATG-3’ |
| **mMMP9** | Forward: 5’- TCCGTGTCCT GTAAATCTGC-3’ |
|  | Reverse: 5’-TCCGTGTCCTGTAAATCTGC-3’ |
